# Why unchosen options linger in our minds

**DOI:** 10.1038/s42003-021-02803-w

**Published:** 2021-11-08

**Authors:** Sophie Bavard, Stefano Palminteri

**Affiliations:** 1 Laboratoire de Neurosciences Cognitives et Computationnelles, Institut National de la Santé et Recherche Médicale, 29 rue d’Ulm, 75005 Paris, France; 2 Ecole normale supérieure, 29 rue d’Ulm, 75005 Paris, France; 3 Université de Recherche Paris Sciences et Lettres, 60 rue Mazarine, 75006 Paris, France

## Abstract

In the 1930s, philosopher John Dewey stated: “We do not learn from experience… we learn from reflecting on experience.” The question of how we learn from the consequences of our actions has been investigated for decades. When deliberating between options, it is assumed that the outcome of our choice is used as a feedback signal to learn the value of the chosen option. But what about the forgone alternative? In a recent paper, Biderman and Shohamy show that we also revise the valuation of forgone options, assuming them to be inversely related to that of chosen ones.

When choosing between two options, it is assumed that one has assigned values to each option and compares them when deliberating. Over the years, research has shown that the outcome of our choice is used to update our ‘value’ of the chosen option. What has remained elusive however, is whether and how we also infer outcomes for unchosen options.

A recent study from Biderman and Shohamy^1^ investigates the mechanism that allows unchosen options to linger in our minds and the consequences of such lingering for their perceived value. Over 600 participants acted as art dealers choosing between pairs of paintings. In a first phase of the experiment, the chosen paintings were virtually sold in a fake auction and could either make a profit (rewarded) or not (unrewarded). Importantly, the outcome of the unchosen painting was not revealed. In a second phase, the pairs of paintings were reordered and participants were asked to determine the most valuable painting out of each pair. Unbeknownst to them, these reordered pairs were either two previously chosen paintings (which had already gone through auction) or two previously unchosen paintings. Finally, the authors tested participants’ memory of the original pairs of paintings they had chosen from in the first phase. Results show that participants value unchosen paintings negatively if they were previously compared with a rewarded painting, and positively when previously compared with an unrewarded painting. In other words, the inferred value transfers in the opposite direction of the learned value, thus increasing the difference in value between the options. In addition, results from the third phase show that the more participants remembered the original pairs, the stronger this contrast became. Together, these findings suggest that deliberation binds choice options together in memory, such that the learned value of one can affect the inferred value of the other.

This study provides evidence that exposure to the outcomes of our chosen option prompts a counterfactual inference about the value of the unchosen one. This inference is related to memory, as stronger memory for the deliberated options is related to a stronger contrast between the values assigned to chosen and unchosen options. The association highlighted by Biderman and Shohamy parallels recent studies showing that counterfactual thinking and memory share a common brain network, including the hippocampal area. As a brain region crucial for memory, the hippocampus may play an important role in these associations. The implications of impaired hippocampal function on such countefactual inferences remain to be investigated.
